# BACE inhibitor treatment of mice induces hyperactivity in a Seizure-related gene 6 family dependent manner without altering learning and memory

**DOI:** 10.1038/s41598-021-94369-0

**Published:** 2021-07-23

**Authors:** A. Nash, H. J. M. Gijsen, B. J. Hrupka, K. S.-L. Teng, S. F. Lichtenthaler, H. Takeshima, J. M. Gunnersen, K. M. Munro

**Affiliations:** 1 Department of Anatomy and Physiology, University of Melbourne, Melbourne, VIC Australia; 2 Discovery Sciences, Janssen Research & Development, Beerse, Belgium; 3 Department of Neuroscience, Janssen Research & Development, Beerse, Belgium; 4 German Centre for Neurodegenerative Diseases (DZNE), Munich, Germany; 5 Neuroproteomics, School of Medicine, Klinikum rechts der Isar, Technical University of Munich, 81675 Munich, Germany; 6 Munich Cluster for Systems Neurology (SyNergy), Munich, Germany; 7 Division of Pharmaceutical Sciences, Graduate School and Faculty of Pharmaceutical Sciences, Kyoto University, Kyoto, Japan; 8 The Florey Institute of Neuroscience and Mental Health, University of Melbourne, Melbourne, VIC Australia

**Keywords:** Learning and memory, Motor control, Neuroscience, Spine structure

## Abstract

BACE inhibitors, which decrease BACE1 (β-secretase 1) cleavage of the amyloid precursor protein, are a potential treatment for Alzheimer’s disease. Clinical trials using BACE inhibitors have reported a lack of positive effect on patient symptoms and, in some cases, have led to increased adverse events, cognitive worsening and hippocampal atrophy. A potential drawback of this strategy is the effect of BACE inhibition on other BACE1 substrates such as Seizure-related gene 6 (Sez6) family proteins which are known to have a role in neuronal function. Mice were treated with an in-diet BACE inhibitor for 4–8 weeks to achieve a clinically-relevant level of amyloid-β40 reduction in the brain. Mice underwent behavioural testing and postmortem analysis of dendritic spine number and morphology with Golgi-Cox staining. Sez6 family triple knockout mice were tested alongside wild-type mice to identify whether any effects of the treatment were due to altered cleavage of Sez6 family proteins. Wild-type mice treated with BACE inhibitor displayed hyperactivity on the elevated open field, as indicated by greater distance travelled, but this effect was not observed in treated Sez6 triple knockout mice. BACE inhibitor treatment did not lead to significant changes in spatial or fear learning, reference memory, cognitive flexibility or anxiety in mice as assessed by the Morris water maze, context fear conditioning, or light–dark box tests. Chronic BACE inhibitor treatment reduced the density of mushroom-type spines in the somatosensory cortex, regardless of genotype, but did not affect steady-state dendritic spine density or morphology in the CA1 region of the hippocampus. Chronic BACE inhibition for 1–2 months in mice led to increased locomotor output but did not alter memory or cognitive flexibility. While the mechanism underlying the treatment-induced hyperactivity is unknown, the absence of this response in Sez6 triple knockout mice indicates that blocking ectodomain shedding of Sez6 family proteins is a contributing factor. In contrast, the decrease in mature spine density in cortical neurons was not attributable to lack of shed Sez6 family protein ectodomains. Therefore, other BACE1 substrates are implicated in this effect and, potentially, in the cognitive decline in longer-term chronically treated patients.

## Introduction

BACE inhibitors, which decrease BACE1 (β-secretase) cleavage of the amyloid precursor protein (APP) and subsequently reduce neurotoxic amyloid-β (Aβ) levels, have been in clinical trials for the treatment of Alzheimer’s disease. BACE inhibitor (BACEi) treatment has shown no positive effect on cognition in patients^1–4^ and has led to increased cognitive decline^2,3,5^, more hippocampal atrophy^1,2,4,6^ and increased adverse events including sleep disturbance, anxiety and weight loss^1,2,7^. The causes of the negative outcomes associated with BACE inhibition have not been determined but may relate to altered proteolytic cleavage of the numerous non-APP BACE1 substrates in the brain^8–10^, echoing issues seen with the use of γ-secretase inhibitors^11^. The Seizure-related gene 6 (Sez6) family of proteins: Sez6, Sez6-like (Sez6L) and Sez6-like 2 (Sez6L2), are BACE1 substrates^10,12,13^ with roles in neuronal development and function^14–18^. Altered proteolytic cleavage of Sez6 contributes to the reversible alterations in synaptic plasticity seen in mice treated with BACEi^19,20^. In this study, we have examined the effect of chronic BACEi treatment on mouse behaviour and dendritic spine properties and determined whether the observed effects are related to altered Sez6 family proteolytic cleavage by comparing wild-type (WT) and Sez6 family triple knockout (TKO) mice.

## Materials and methods

### Mice and drug administration

**Figure 1:**
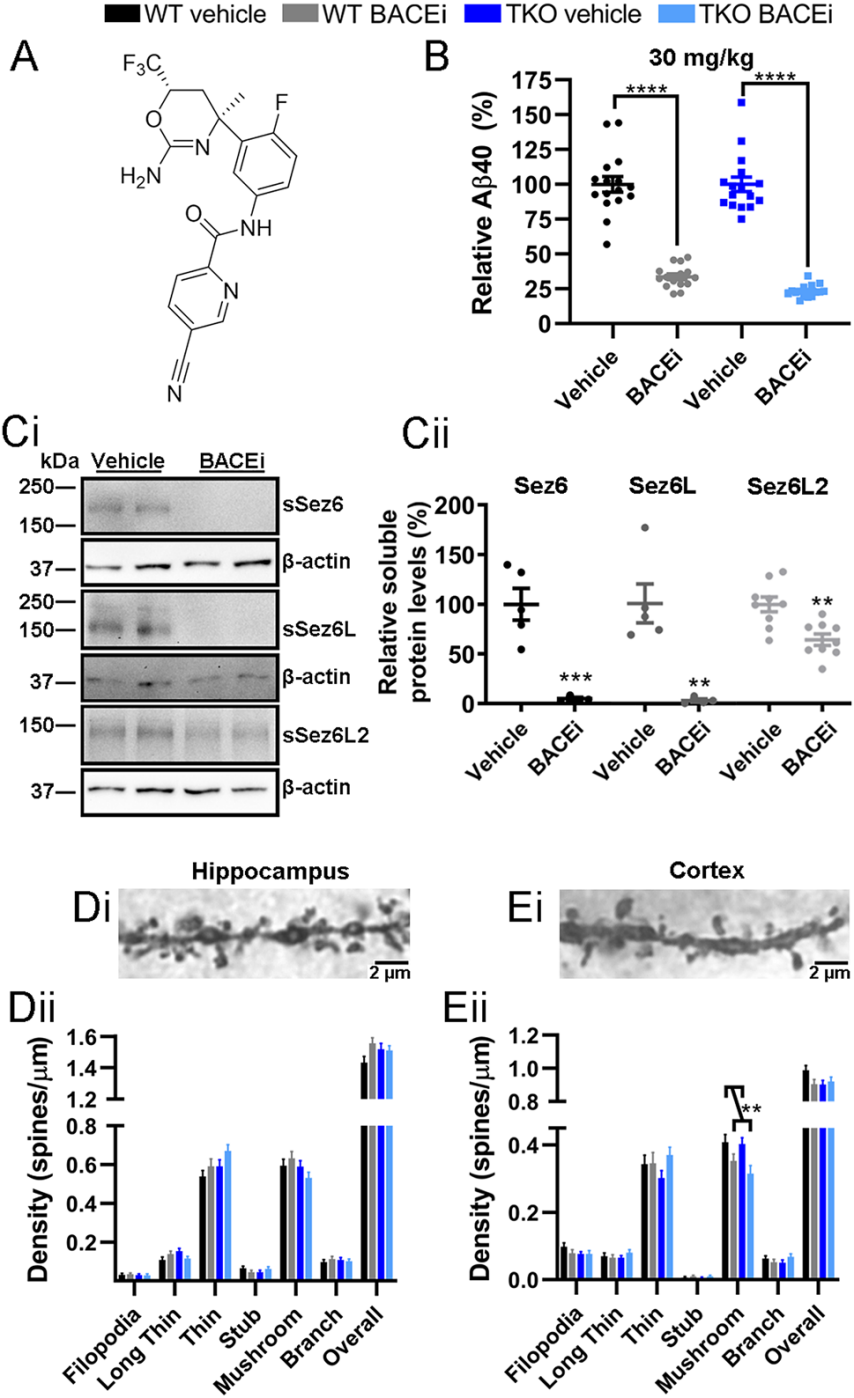
BACEi treatment decreases Aβ40 and Sez6 family protein ectodomain levels in the mouse brain and reduces cortical mushroom spine density. (**A**) The molecular structure of BACEi Compound H. (**B**) Aβ40 levels after 30 mg/kg/day BACEi treatment are reduced in WT and Sez6 TKO brains by 67% and 77% respectively as detected by Meso Scale Discovery assay. n = 15–16 per group; 2-way ANOVA: genotype p = 0.21, treatment p < 0.0001, interaction p = 0.21. (**C**) Sez6, Sez6L and Sez6L2 ectodomain protein fragments in brains from BACEi treated WT mice were decreased by 95%, 96% and 36% respectively compared to vehicle treated WTs as detected by Western blot. Representative Western blot (**i**) and quantitation of all samples (**ii**). n = 4–9/treatment; unpaired t-test: Sez6 p = 0.0012, Sez6L p = 0.0032, Sez6L2 p = 0.0018. Full-length blots are presented in Supplementary Figs. 2–4. (**Di**) Representative image of hippocampal dendrite segment used for analysis of dendritic spines. (Image shows a single Z plane however individual spines were examined in multiple planes as described in “Methods”). (**Dii**) BACEi treatment did not affect the density of dendritic spines on oblique apical secondary branches of hippocampal CA1 pyramidal neurons. The overall density and densities of individual spine classes were not altered by treatment in either WT or Sez6 TKO mice. n = 35 neurons (from 7 brains) per genotype/treatment; 2-way nested ANOVA of overall density: genotype p = 0.71, treatment p = 0.18, interaction p = 0.16. (**Ei**) Representative image of a cortical dendrite segment used for analysis of dendritic spines. (**Eii**) BACEi treatment did not affect the overall density of dendritic spines on basal secondary dendrites of layer V pyramidal neurons in the somatosensory cortex. However, BACE inhibition specifically reduced mushroom spine density in both WT and Sez6 TKO cortical neurons. WT vehicle: 0.41 ± 0.02 spines/µm, WT BACEi: 0.35 ± 0.02, TKO vehicle: 0.4 ± 0.02, TKO BACEi: 0.32 ± 0.02. n = 35 neurons (from 7 brains) per genotype/treatment; 2-way nested ANOVA of overall density: genotype p = 0.27, treatment p = 0.3, interaction p = 0.11; mushroom spine density: genotype p = 0.4, treatment p = 0.01, interaction p = 0.56. All graphs show mean ± SEM. **p ≤ 0.01, ***p ≤ 0.001, ****p ≤ 0.0001.

Male mice with targeted deletions of the genes encoding Sez6, Sez6L and Sez6L2 (Sez6 TKO^14^) and WT mice were maintained on a predominantly C57BL6 genetic background. Mice were housed under standard conditions and experiments were performed in the daytime. All animal experiments were approved by the Animal Ethics Committee at the University of Melbourne. BACE inhibitor Compound H (*N*-[3-[(4S,6S)-2-amino-5,6-dihydro-4-methyl-6-(trifluoromethyl)-4H-1,3-oxazin-4-yl]-4-fluorophenyl]-5-cyano-2-pyridinecarboxamide; structure shown in Fig. 1A; compound 89 in Ref.^21^ with ~ 4 × higher affinity for BACE1 over BACE2—H. Gijsen, personal communication) was provided by Janssen Pharmaceutica (Beerse, Belgium). BACEi was dissolved in an emulsion of 30% (v/v) peanut oil in MilliQ water. This mixture was combined with irradiated powdered diet (SF14-156; Specialty Feeds, Australia) and made into a dough ball. The amount of BACEi equated to approximately 15 mg/kg body weight (2 g ball) or 30 mg/kg body weight (4 g ball). Mice in the 15 mg/kg/day BACEi group preferentially ate the dough ball and had access to regular chow. Mice in the 30 mg/kg/day group had no or very limited access to regular chow (depending on the individual mouse weight) and all mice included in the study consumed the entirety or the majority of the dough ball each day.

### Experimental timeline

Mice were singly housed from 8 weeks of age. BACEi Compound H was delivered in diet between 8 and 16 weeks of age. Behavioural testing began after 4 weeks of BACEi treatment and was conducted during the remaining 4 weeks of BACEi administration. Mice had at least one day of rest in between behavioural tests. 15 mg/kg/day cohorts underwent testing in the following order: elevated open field, context fear conditioning and extinction and Morris water maze. 30 mg/kg/day cohorts underwent testing in the following order: elevated open field, context fear conditioning and extinction, light/dark box and Morris water maze. On the final day of BACEi treatment, mice were killed with pentobarbitone sodium (Lethabarb, Virbac Australia) and the brain was dissected out and divided into hemispheres, one of which was placed into Golgi-Cox solution for dendritic spine analysis and the other collected for protein analysis.

### Behavioural testing

#### Elevated open field

Mice were placed in the centre of the field, a test arena (75 × 100 cm) without walls positioned 60 cm above the ground, and allowed to roam freely for 3 min. Overhead lighting was switched off and two spotlights on either side of the field shone directly onto the field to create an aversive environment. Videos were obtained and analysed using TopScan Lite (CleverSys Inc., USA).

#### Context fear conditioning and extinction

The fear conditioning chamber was a 25 × 30 × 24 cm plexiglass chamber (CleverSys Inc.) containing a stainless steel shock grid floor. A light cue and visual cues on the chamber walls were used. During the training session, mice explored the chamber for 3 min before receiving 3 × 0.8 mA shocks of 2 s duration with 30 s between each shock. Mice remained in the chamber for an additional 60 s before being transferred back to their home cage. Freezing time was measured for the 3 min pre-shock and 60 s post-shock periods. Mice were placed in the chamber 24 h later and daily thereafter for a further 7 days. In these test and extinction sessions, mice were allowed to explore freely for 3 min without receiving a shock. Fear behaviour (percentage of time spent freezing) was recorded by FreezeScan software (CleverSys Inc.) and analysed by the investigator.

#### Light dark box

Automated locomotor systems (27.5 × 27.5 × 13.5 cm; Med Associates Inc., USA) were used with black Perspex inserts (13.5 × 27.5 cm) to create a dark area in one half of the locomotor activity cell. The light half had a brightness of 950 lx. Mice were placed in the dark side to start the test and locomotor activity over 10 min was recorded and analysed by Activity Monitor software (Med Associates Inc.).

#### Morris water maze

The maze was 1.2 m in diameter and the 10 cm diameter ‘escape’ platform was submerged 1 cm under the surface of the water (20 °C) which was made opaque with white, nontoxic paint. Overhead lighting was switched off and an uplight was placed on top of one of the 3D spatial cues. The arrangement of prominent geometric images, 3D spatial cues and lighting in the region around the pool is known to enable spatial learning in the maze (e.g. Ref.^16^). The investigator was hidden from sight during testing. Each mouse underwent 4 trials per day, starting at one of 4 locations equally spaced around the pool. Each trial lasted a maximum of 120 s and at the end of each trial the mouse was placed or allowed to stay on the platform for 20 s. Acquisition training went for 7 days, during which the location of the platform was kept constant. An automated tracking system (TopScan Lite, CleverySys Inc.) recorded and analysed swim paths. The day after acquisition training was completed, the probe trial took place. In the 30 s probe trial, the platform was removed and each mouse was placed into the pool in a location directly opposite from where the hidden platform had been located. Time spent in the ‘target’ quadrant was used to determine whether mice had learnt the task. After the probe trial, mice began 4 days of reversal training where the hidden platform was moved to a different quadrant. Reversal training trials were conducted as described for acquisition training and at the completion of training each mouse completed a 30 s probe trial.

#### Locomotor cells

Mice were acclimatised to the test room for 1 h immediately prior to testing and were tested for locomotor activity under low lighting conditions (10 lx). Mice were placed in the centre of the locomotor cell (27.5 × 27.5 × 13.5 cm; Med Associates Inc.) and locomotor activity in the horizontal and vertical planes was recorded over a 30 min period.

### Golgi-Cox impregnation

Modified Golgi-Cox impregnation of neurons was performed using the FD RapidGolgi Stain kit (FD NeuroTechnologies Inc., USA). One brain hemisphere from each mouse was fixed, sectioned at 100 µm and developed. Neurons were analysed for dendritic spine properties as described by Risher et al.^22^, with the slight modification that spines with a head width greater than 0.5 µm (rather than 0.6 µm) were classed as mushroom spines, as previously published^16^. Approximately 20 µm lengths of oblique secondary dendrite from neurons in the stratum radiatum of CA1 pyramidal neurons or 30–40 µm lengths of basal secondary dendrites from layer V pyramidal neurons in the somatosensory cortex were selected for analysis. Z-stack images were captured using brightfield on a Zeiss Axio Imager M2 (Zeiss, Germany) using Stereo Investigator (MBF Bioscience, USA). The investigator was blind to the genotype and treatment groups during both imaging and analysis.

### Brain fractionation

Brain hemispheres from mice treated with 30 mg/kg/day BACEi were collected into 1 ml ice cold Dulbecco’s Phosphate Buffered Saline (DPBS, Life Technologies) containing protein inhibitor tablets (cOmplete ULTRA Tablets, Mini, EDTA-free EASYpack Protease Inhibitor Cocktail, Sigma), briefly stored on ice, transferred to 1 ml ice cold diethylamine (DEA) buffer (50 mM NaCl, 2 mM EDTA, 0.2% DEA) for homogenization then underwent fractionation as described in Kuhn et al.^12^, outlined below. Brain hemispheres from mice treated with 15 mg/kg/day BACEi were collected into 1 ml ice cold MilliQ water, briefly stored on ice until all brains were collected, homogenized in MilliQ water and homogenates were stored at − 20 °C. Brain homogenates were thawed on ice and 500 µl of 3 × DEA buffer was added before samples underwent fractionation. Homogenates from all samples were neutralized with 1 M Tris (pH 7.6) and centrifuged at 20,817 RCF at 4 °C for 10 min. The supernatant was collected and spun at 186,000 RCF at 4 °C for 30 min in an ultracentrifuge (Beckman Coulter Inc., USA); this supernatant was collected and stored at − 20 °C as the ‘soluble fraction’. The pellet from the initial 20,817 RCF spin was washed with DPBS, centrifuged at 20,817 RCF then resuspended in 200 µl STE buffer (150 mM NaCl, 50 mM Tris, 2 mM EDTA, 1% Triton X-100) before being centrifuged again at 20,817 RCF at 4 °C for 10 min; the supernatant from this spin was collected and stored as the ‘membrane fraction’.

### Western blot

Protein concentrations were quantified using the DC Protein Assay kit (Bio-Rad, USA) and 40 µg of total protein were used for Western Blot analysis. Samples were boiled for 5 min at 95 °C in Laemmli buffer. For the detection of Sez6L, Laemmli buffer without the disulfide bridge reducing agent β-mercaptoethanol was used and samples were not boiled^13^. Samples were separated on 7.5% Mini-PROTEAN TGX Stain-Free gels (Bio-Rad). Nitrocellulose or PVDF membranes (Bio-Rad) were incubated with primary antibody overnight at 4 °C. After incubation with secondary antibody at room temperature for 1 h, membranes were developed with Clarity ECL (Bio-Rad). The following antibodies were used: monoclonal Sez6 (1:100; see Ref.^13^), monoclonal Sez6L (1:100; Ref.^13^), polyclonal Sez6L2 (1:500; R&D Systems, AF4916), β actin (1: 1000; Sigma, A5441) and monoclonal APP (Y188; 1:6000; Abcam, ab32136), HRP coupled anti-rat, anti-rabbit, anti-goat and anti-mouse (Life Technologies). Western blot protein levels were normalized to total blot protein using BioRad stain-free technology and the ChemiDoc imager as described^23^.

### Meso Scale Discovery detection of Aβ40

The concentration of Aβ40 in the membrane fraction of brains from BACEi and vehicle treated mice was determined using the V-PLEX Aβ40 Peptide Panel (Meso Scale Diagnostics, USA) as per manufacturer’s instructions. Two technical replicates of each sample were run. Concentration in pg/ml was converted to pg/µg using the total protein concentration of the samples as determined by the DC Protein Assay kit (see above).

### Cocaine treatment

Mice were administered either 20 mg/kg cocaine or saline by intraperitoneal injection immediately prior to undergoing testing on the elevated open field and locomotor cell (as described above). Mice were tested first on the elevated open field then in the locomotor cell with at least 3 days break between tests.

### Statistical analyses

Context fear conditioning and Morris water maze data were analysed using a two-way ANOVA with repeated measures and the elevated open field, light/dark box and locomotor cell data were analysed using a 2-way ANOVA. Aβ40 concentration was analysed using a 2-way ANOVA and normalized Western blot protein levels were analysed using an unpaired t-test. As indicated by Box’s test of equality of covariance matrices, Morris water maze pathlength and latency data were transformed with a log_10_ transformation. If Mauchly’s test of sphericity was determined to be significant, the Greenhouse–Geisser correction was used (applicable to Morris water maze and context fear conditioning data). Hippocampal and cortical spines were analysed using a 2-way nested ANOVA. The Bonferroni post-hoc test was used for all relevant analyses. All data are represented as mean ± standard error of the mean (SEM), except for the Morris water maze probe trials which are represented as mean ± 95% confidence interval (as per Rogers et al.^24^). Results were considered statistically significant when a p < 0.05 was obtained. Data were analysed in GraphPad Prism 7 (GraphPad Software Inc., USA), Minitab 18 or IBM SPSS Statistics 25 (IBM Corporation, USA).

### Ethics approval and consent to participate

All animal experiments were approved by the Animal Ethics Committee (Anatomy & Neuroscience, Pathology, Pharmacology & Therapeutics and Physiology) at the University of Melbourne and conducted in accordance with the National Health & Medical Research Council Australian Code for the Care and Use of Animals for Scientific Purposes. This study was carried out in compliance with the ARRIVE guidelines.

### Consent for publication

Not applicable.

## Results

### Chronic BACEi treatment decreases Aβ40 and Sez6 family protein ectodomain levels in the mouse brain and decreases mushroom spine density in the cortex

Mice treated daily for 2 months in total with ~ 30 mg Compound H per kg body weight (structure in Fig. 1A) had a substantial decrease in Aβ40 concentration in brain tissue compared to vehicle treated mice, equating to a 67 and 77% decrease in WT and Sez6 TKO brains respectively (Fig. 1B). This level of BACE inhibition markedly decreased the levels of soluble Sez6 family protein ectodomains in WT samples (Fig. 1C), particularly Sez6 and Sez6L (95% and 96% reduction respectively), previously shown to be prominent BACE1 substrates^12,13^, and Sez6L2 (36% reduction). Chronic BACE inhibition did not result in changes to dendritic spine number or morphology in the hippocampus (Fig. 1D) however it decreased mushroom spine density in the somatosensory cortex in both WT and Sez6 TKO mice (Fig. 1E). There was a tendency for overall spine density to be reduced in BACEi vs. vehicle-treated WT brains but this was not significant at an α of 0.05 (Fig. 1E). There was no effect of BACEi treatment on spine length, width or length:width ratio in the hippocampus or cortex (Supplementary Fig. 1A,B). Although there was a higher level of Aβ40 in vehicle treated Sez6 TKO compared to WT samples (Supplementary Fig. 1C), this was not apparent in a separate 15 mg/kg/day cohort using a different sequential extraction protocol (Supplementary Fig. 1D). There was no significant difference in full-length APP levels between genotypes in either cohort as determined by Western blot (Supplementary Fig. 1E,F). Mice treated with 15 mg/kg/day BACEi showed a 54–55% reduction in Aβ40 (Supplementary Fig. 1D), a 75% reduction in soluble Sez6 (not shown) and no significant change in soluble Sez6L2 (not shown).

### BACEi treatment does not alter spatial memory or cognitive flexibility in mice

**Figure 2:**
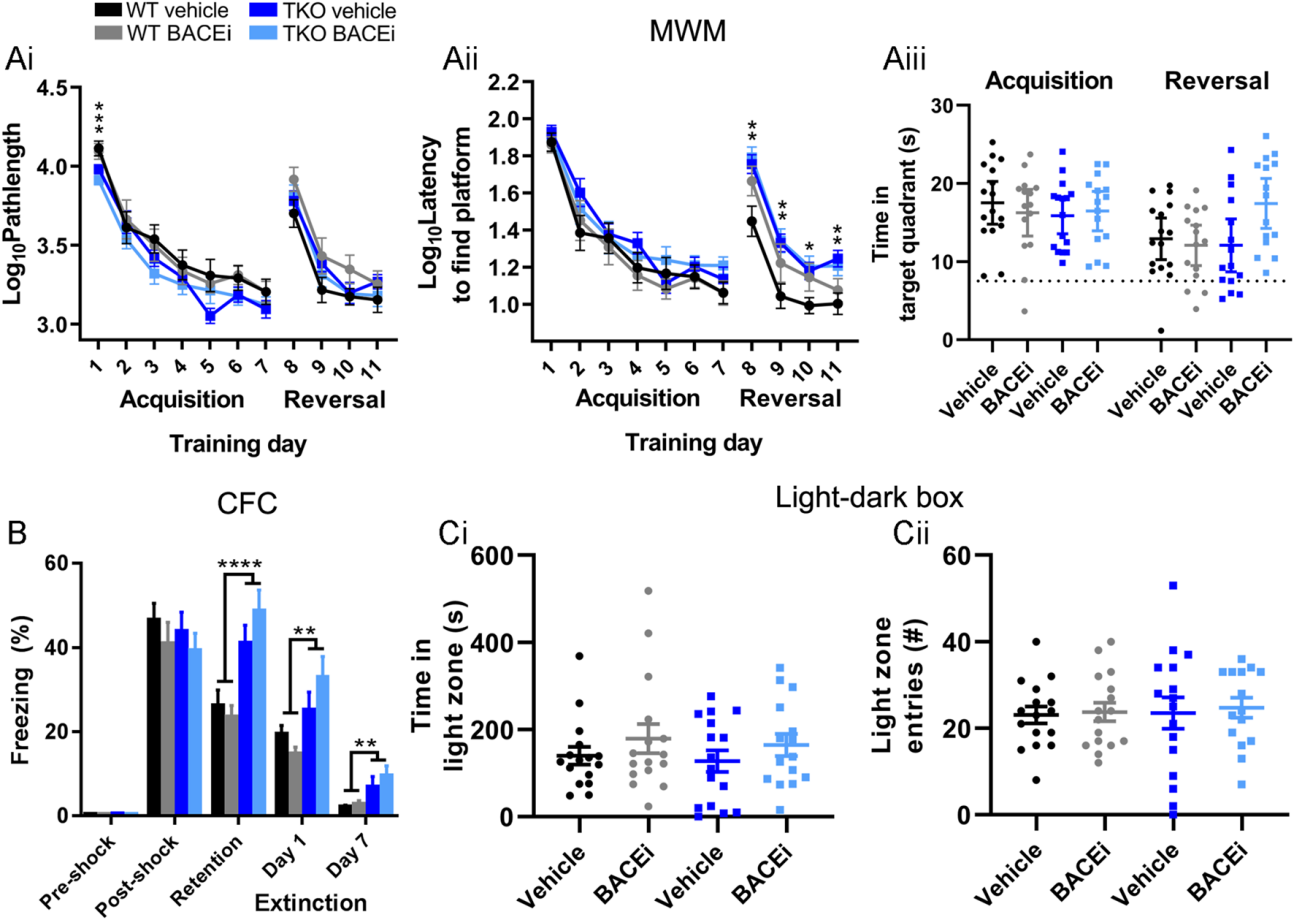
BACEi treatment does not alter spatial memory or cognitive flexibility in mice. (**A**) In the Morris water maze (MWM), WT and Sez6 TKO mice swam an equivalent distance to reach the hidden platform during acquisition training (from day 2) and reversal training (**i**) and Sez6 TKOs took a longer time than WT mice to find the hidden platform during reversal training (**ii**). 30 mg/kg/day BACEi treatment did not significantly affect pathlength or latency within genotypes (**i–ii**), or the ability of mice to successfully learn the hidden platform location in the acquisition and reversal probe trials (**iii**) although a tendency for BACEi treated WT mice to take longer to find the platform than vehicle treated WT mice on the first day of reversal training is noted (**ii**). n = 15–16 per group. (**Ai**) Log_10_ of pathlength (mm) and (**Aii**) Log_10_ of latency (seconds): 2-way RM ANOVA with acquisition and reversal analysed separately. (**Ai**) Acquisition: genotype p = 0.015, day p < 0.0001, treatment p = 0.73. (**Ai**) Reversal: genotype p = 0.891, day p < 0.0001, treatment p = 0.206. (**Aii**) Acquisition: genotype p = 0.091, day p < 0.0001, treatment p = 0.855. (**Aii**) Reversal: genotype p = 0.0004, day p < 0.0001, treatment p = 0.095. No significant interactions observed. Stars indicate a significant Bonferroni post-hoc at the level of genotype × day effect (treatment groups pooled). Aiii: 95% CI did not overlap with chance (7.5 s) for any group. (**B**) Sez6 TKO mice had enhanced fear learning at the 24 h context fear conditioning (CFC) retention test and delayed fear extinction compared to WT mice, but behaviour within each genotype was unaffected by 30 mg/kg/day BACEi treatment. n = 15–16 per group. 2-way RM ANOVA: genotype p < 0.0001, timepoint p < 0.0001, treatment p = 0.96, genotype × timepoint p < 0.0001, no other significant interactions. Stars indicate a significant Bonferroni post-hoc at the level of genotype × timepoint effect. (**C**) In the light/dark box test of anxiety, WT and Sez6 triple KO mice treated with 30 mg/kg/day BACEi spent an equivalent amount of time in the light zone (**i**) and made an equivalent number of entries into the light zone (**ii**). n = 15–16 per group. 2-way ANOVA. (**Ci**) genotype p = 0.62, treatment p = 0.16. (**Cii**) genotype p = 0.79, treatment p = 0.72. No significant interactions. All graphs show mean ± SEM except (**Aiii**) which shows mean ± 95% CI. *p ≤ 0.05, **p ≤ 0.01, ***p ≤ 0.001, ****p ≤ 0.0001.

Mice underwent behavioural testing between weeks 4–8 of daily BACEi treatment. In the Morris water maze, mice treated with 30 mg/kg/day Compound H learnt the platform location during the acquisition phase in the same manner as vehicle treated mice (Fig. 2Ai,ii) and during the probe trials (testing memory of the platform location) they spent the same amount of time in the target quadrant as vehicle treated controls (Fig. 2Aiii). Sez6 TKO mice displayed deficits in cognitive flexibility, taking a longer time to locate the hidden platform during the reversal phase (Fig. 2Aii) as previously described^16^. However, within genotypes, BACEi treatment did not significantly change the pathlength or latency to find the platform in the reversal phase (Fig. 2Ai,ii); BACEi treated WT mice tended to take longer to find the platform than vehicle treated WT mice on the first day of reversal training but this was not significant at an α of 0.05 (Fig. 2Aii). Similar results were observed in mice treated with 15 mg/kg/day BACEi (not shown), with no effect of treatment on Morris water maze performance. During context fear conditioning, Sez6 TKO mice displayed enhanced freezing (Fig. 2B) in accordance with previous results^16^. However, within genotypes, 30 mg/kg/day BACEi treatment did not affect learning or extinction associated with context fear conditioning (Fig. 2B). Similar results were observed in mice treated with 15 mg/kg/day BACEi (not shown). 30 mg/kg/day BACEi and vehicle treated mice spent the same amount of time in the light zone in the light/dark box, a test of anxiety (Fig. 2Ci,ii) and there was additionally no effect of genotype in this test.

### BACEi treatment increases hyperactivity on the elevated open field in a Sez6 family protein dependent manner

**Figure 3:**
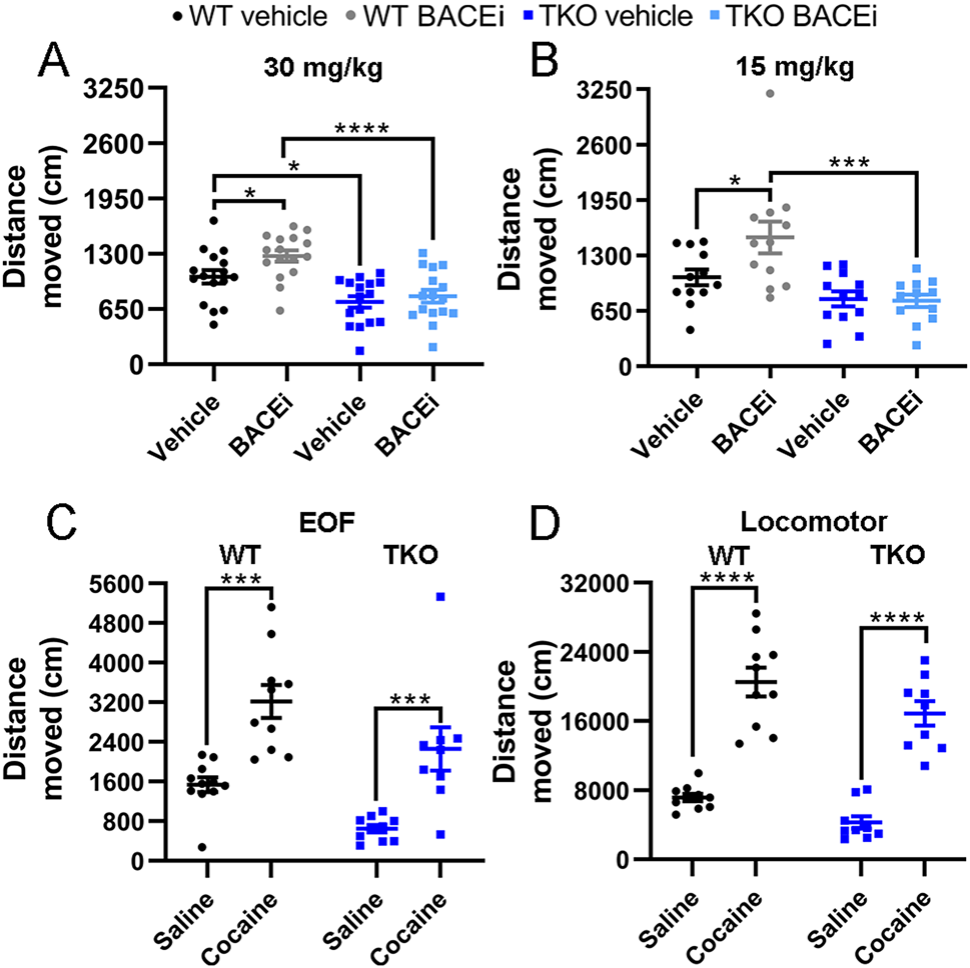
BACEi treatment increases hyperactivity on the elevated open field in a Sez6 family protein dependent manner. (**A**) WT mice treated with 30 mg/kg/day BACEi moved a greater distance on the elevated open field (EOF) than vehicle treated mice, but this increased movement was not observed in BACEi treated Sez6 TKO mice. n = 16 per group. 2-way ANOVA: genotype p < 0.0001, treatment p = 0.036, interaction p = 0.21. Bonferroni post-hoc analysis: WT vehicle vs. WT BACEi p = 0.038, Sez6 TKO vehicle vs. Sez6 TKO BACEi p > 0.99. (**B**) WT mice treated with 15 mg/kg/day BACEi moved a greater distance on the EOF than vehicle treated mice, but this increased movement was not observed in BACEi treated Sez6 TKO mice. n = 12 per group. 2-way ANOVA: genotype p = 0.0001, treatment p = 0.067, interaction p = 0.045. Bonferroni post-hoc analysis: WT vehicle vs. WT BACEi p = 0.016, Sez6 TKO vehicle vs. Sez6 TKO BACEi p > 0.99. (**C**) A 20 mg/kg body weight intraperitoneal injection of cocaine immediately before testing on the EOF increased the distance travelled by WT and Sez6 TKO by 110% and 254% respectively. n = 9–11 per group. 2-way ANOVA: genotype p = 0.0017, treatment p < 0.0001, interaction p = 0.89. (**D**) Cocaine treatment before testing in locomotor cells increased the distance travelled by WT and Sez6 TKO by 187% and 281% respectively. n = 9–10 per group. 2-way ANOVA: genotype p = 0.0087, cocaine treatment p < 0.0001, interaction p = 0.75. Stars indicate a significant Bonferroni post-hoc analysis. All graphs show mean ± SEM. *p ≤ 0.05, ***p ≤ 0.001, ****p ≤ 0.0001.

On the elevated open field, a test of anxiety and exploration^25^, WT mice treated with 30 mg/kg/day Compound H moved significantly more than vehicle treated WT mice (Fig. 3A), representing an increase of ~ 24%. Vehicle treated Sez6 TKO mice moved less than vehicle treated WTs, in agreement with previous results^16^. Sez6 TKO mice treated with 30 mg/kg/day BACEi moved the same amount as vehicle treated Sez6 TKO mice (Fig. 3A). Similar results were seen with a lower dose of 15 mg/kg/day BACEi: WT mice moved ~ 45% more when treated with BACEi while the movement of BACEi treated Sez6 TKO mice was unchanged by drug treatment (Fig. 3B). The significant genotype × drug interaction (p = 0.045) suggests that the hyperactivity caused by BACEi treatment is dependent on decreased BACE1 mediated ectodomain shedding of Sez6 family proteins. Sez6 TKO mice are known to exhibit motor deficits which affect their motor learning and co-ordination^16^, so to support this interpretation it was important to determine whether Sez6 TKOs were capable of responding to a psychomotor stimulant by substantially increasing their distance travelled on the elevated open field. In response to 20 mg/kg cocaine treatment, both WT and Sez6 TKO mice showed a considerable increase in movement on the elevated open field (110% and 254% respectively; Fig. 3C) and in locomotor cells (187% and 281%; Fig. 3D) indicating that the differential response of WT and Sez6 TKO mice to BACEi treatment is not solely due to deficits in motor capabilities.

## Discussion

The clearest effect of BACEi treatment on mouse behavior observed in this study, hyperactivity in an aversive environment, is likely due to reduced cleavage of Sez6 family proteins, adding support to the concept that interference with certain functions of one or more Sez6 family members contributes to the negative outcomes seen with BACE inhibition in patients. These effects may be mediated through accumulation of Sez6 family protein transmembrane isoforms and/or by the reduction of shed Sez6 family ectodomains. While the significance of BACEi associated hyperactivity in mice is unclear, heightened locomotor activity has been correlated with increased hippocampal activity in APP transgenic mice (reviewed by Refs.^26,27^) and associated with dendritic spine instability^28^, suggesting the hyperactivity may reflect the early stages of hippocampal dysfunction. The behavioural effects of BACEi treatment in mice presented here are similar to those reported for a conditional knockout line with BACE1 deletion in postnatal forebrain excitatory neurons^29^, namely hyperactivity on the open field, indicated by greater distance travelled, and no memory impairment in context fear conditioning or in the probe trial of the Morris water maze. Whilst no memory impairment in context fear conditioning was also observed in an adult conditional knockout with partial BACE1 deletion^30^, both this study and a previous study of BACE inhibition in mice^19^ report no hyperactivity on the open field. Results from the former are in contrast to our findings from the 15 mg/kg/day BACEi cohort, with both protocols causing an ~ 50% decrease in Aβ40 brain levels in adult mice (Ref.^30^, Supplementary Fig. 1D). The unwalled, elevated open field used in this study is a slightly more aversive environment than a standard open field protocol which may contribute to these conflicting findings. Future studies should examine the effect of different BACEi drugs on hyperactivity in rodents to establish whether this behavioural change is consistently observed, and clarify whether the differential effect of BACEi treatment on hyperactivity in Sez6 TKO and control mice is dosage dependent. Compound H should also be tested in BACE1 KO mice to identify any BACE1-independent effects. Future studies could also examine whether hyperactivity is seen in single Sez6 or Sez6L gene knockout mice treated with BACEi; altered cleavage of Sez6L2 is unlikely to be a major contributing factor as hyperactivity was seen in mice treated with a BACEi dosage (15 mg/kg/day) that did not significantly alter Sez6L2 shedding. Alternatively, a BACE1-resistant Sez6 or Sez6L knock-in mouse model would enable examination of altered Sez6 cleavage without requiring total gene knockout and without causing the concurrent alteration of other BACE1 substrates that occurs with BACEi treatment.

Delayed learning in the Morris water maze was observed in older BACE1 conditional knockouts^29^ but not in BACEi treated mice in this study, suggesting that learning deficits might become apparent after a longer BACEi treatment period and/or in older mice. It is possible that more subtle cognitive changes occurring in human patients would not be easily seen in BACEi treated mice in our test paradigms; mouse touchscreen test batteries modelling patient cognitive examinations^31,32^ could be incorporated into future preclinical studies.

BACEi treatment in mice is known to alter dendritic spine turnover^19^ in a reversible and Sez6-dependent manner^20^ therefore it was important to determine whether BACE inhibition in this study produced similar changes in spine number or classification to those seen in Sez6 knockout^15^ and Sez6 TKO^16^ mice. Similar to previous reports of BACEi-induced decreases in spine numbers on apical dendrites of cortical neurons^19,20,33^, BACEi treatment reduced mushroom spine density on basal dendrites of pyramidal cortical neurons. In this case, the reduction was not Sez6 family member dependent, as a similar decrease was observed in neurons from BACEi treated WT and Sez6 TKO mice. This observation might indicate opposing effects of the shed ectodomains of Sez6L/Sez6L2 and Sez6 on mushroom spine density and this possibility could be explored further in the single gene knockout lines. It is also relevant that the effect of BACE inhibition on cognition was tested prior to taking the brains for dendritic spine analyses and, thus, mice had experienced environmental enrichment during behavioural testing. Enrichment alters spine properties including density^34^, which may have masked any treatment-induced changes and the previously identified shift from mature to immature spines seen in behaviourally naïve Sez6 TKO mice^16^. Nevertheless, our results reinforce the need to examine the effect of chronic BACEi treatment on spine density and morphology with particular attention given to mushroom spine types, noting that any spine changes will vary according to neuron and dendrite type. The recently reported hippocampal long-term potentiation deficit in mice with only partial adult BACE1 deletion^30^ emphasizes the importance of understanding how synaptic plasticity may be affected in patients treated with BACE inhibitors.

## Conclusions

The recent approval of Aβ antibody aducanumab for the treatment of Alzheimer’s disease^35^ may increase enthusiasm for BACE inhibitors as a complementary therapeutic approach despite their past failures in clinical trials. It is therefore important to clearly identify the factors underlying the increased cognitive worsening seen in BACEi clinical trials. While lowering the dosage of BACE inhibitors may limit the negative effects of treatment on cognition^2,3^, modifications which specifically reduce the impact of treatment on key BACE1 substrates such as Sez6 family proteins should be considered in order to maximise the potential benefit of this strategy. In this report, no significant deficits in memory or cognitive flexibility in mice treated with BACEi were observed, suggesting that clinical results may relate to the age, disease state and/or duration of treatment. However, even in young mice, BACEi increased locomotor activity in an aversive environment and this effect depended on Sez6 family proteins, providing evidence that interfering with Sez6 protein functions contributes to the adverse effects seen with BACE inhibition.

## Supplementary Information

Supplementary Figures.

## Data Availability

All data generated and analysed during this study are included in this published article.

## Abbreviations

Aβ: Amyloid-β
ANOVA: Analysis of variance
APP: Amyloid precursor protein
BACE1: β-Secretase 1; β-site amyloid precursor protein cleaving enzyme 1
BACEi: BACE inhibitor
CFC: Context fear conditioning
CI: Confidence interval
DEA: Diethanolamine
DPBS: Dulbecco’s phosphate buffered saline
EOF: Elevated open field
MSD: Meso Scale Discovery
MWM: Morris water maze
RM: Repeated measures
SEM: Standard error of the mean
Sez6: Seizure-related gene 6
Sez6L: Seizure-related gene 6-like
Sez6L2: Seizure-related gene 6-like 2
TKO: Triple knockout
WT: Wild-type
