# Supplementary material for: BACE inhibitor treatment of mice induces hyperactivity in a Seizure-related gene 6 family dependent manner without altering learning and memory
Source: Sci Rep. 2021 Jul 23;11:15084. doi: 10.1038/s41598-021-94369-0 (PMC8302682; doi:10.1038/s41598-021-94369-0)
Supplement: Supplementary file 1 — Supplementary Figures. [file 41598_2021_94369_MOESM1_ESM.pdf]

Supplementary File

BACE inhibitor treatment of mice induces hyperactivity in a Seizure-related gene 6 family dependent manner without altering learning and memory

A. Nash, H. J. M. Gijsen, B. J. Hrupka, K. S-L. Teng, S. F. Lichtenthaler, H. Takeshima, J. M. Gunnersen, K. M. Munro

Supplementary Figure 1

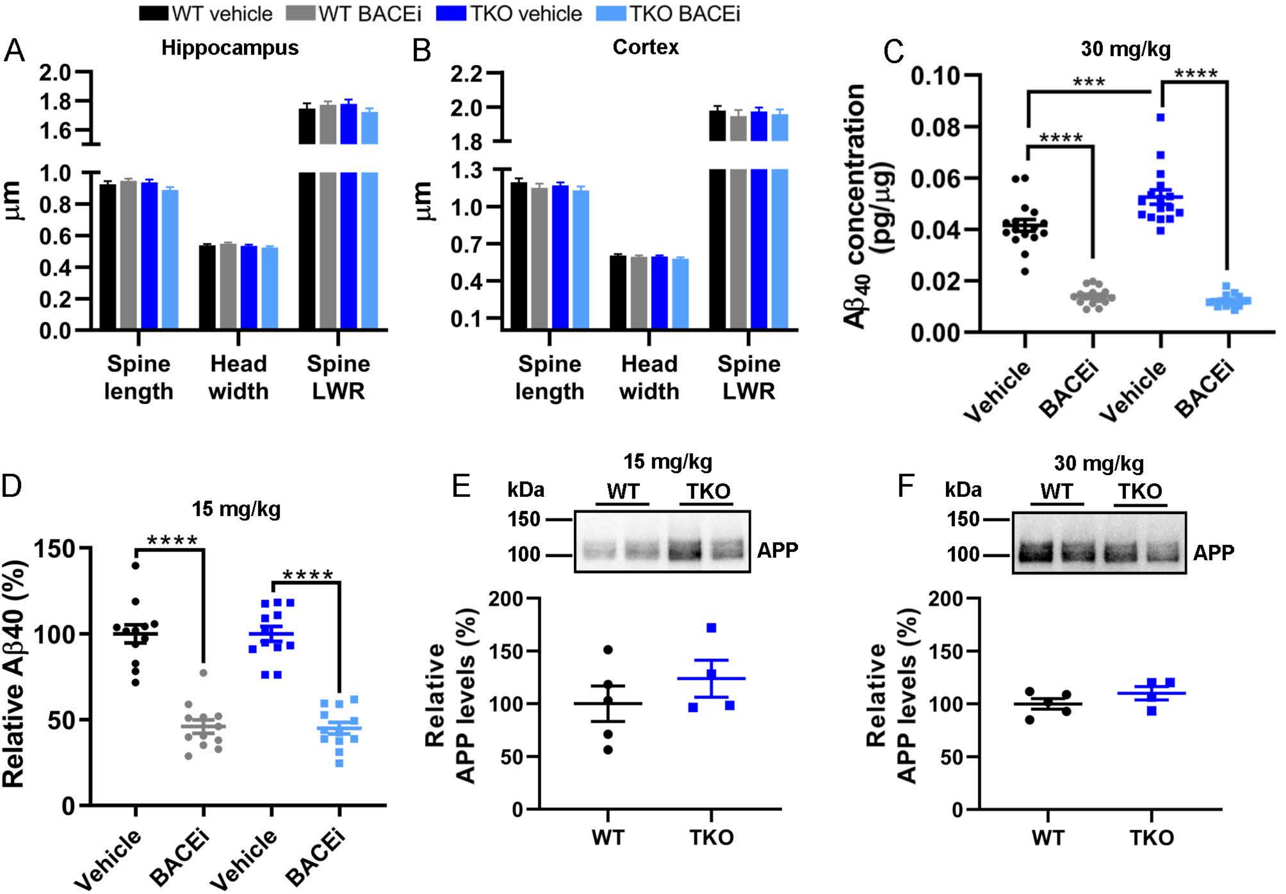

Supplementary Figure 1.

A, B) The length and width of dendritic spines on hippocampal (B) and cortical (C) neurons was unchanged by BACEi treatment. n=35 neurons (from 7 brains) per genotype/treatment, 2-way nested ANOVA. Analysis of hippocampal spine length, hippocampal spine width, cortical spine length and cortical spine width did not show a significant effect of genotype, treatment or interaction ($p > 0.05$). **C)** Aβ40 concentration after eight weeks of 30mg/kg/day BACEi treatment are reduced in WT and Sez6 TKO brains as detected by MSD assay. n=15-16 per group; 2-way ANOVA genotype p=0.019, treatment p<0.0001, interaction p=0.0016; groups compared with Bonferroni post-hoc analysis. **D)** Aβ40 levels after eight weeks of 15mg/kg/day BACEi treatment are reduced in WT and Sez6 TKO brains by 54% and 55% respectively as detected by MSD assay. n=12 per group; 2-way ANOVA: genotype p=0.55, treatment p<0.0001, no significant interaction. **E)** Levels of full-length APP in the brain did not differ between vehicle treated WT and Sez6 TKO mice in the 15mg/kg/day cohort as detected by Western blot. N=4-5 per group. Unpaired t test p=0.37. Full-length blots are presented in Supplementary Figure 2. **F)** Levels of full-length APP in the brain did not differ between vehicle treated WT and Sez6 TKO mice in the 30mg/kg/day cohort as detected by Western blot. N=4-5 per group. Unpaired t test p=0.99. Full-length blots are presented in Supplementary Figures 5-6.

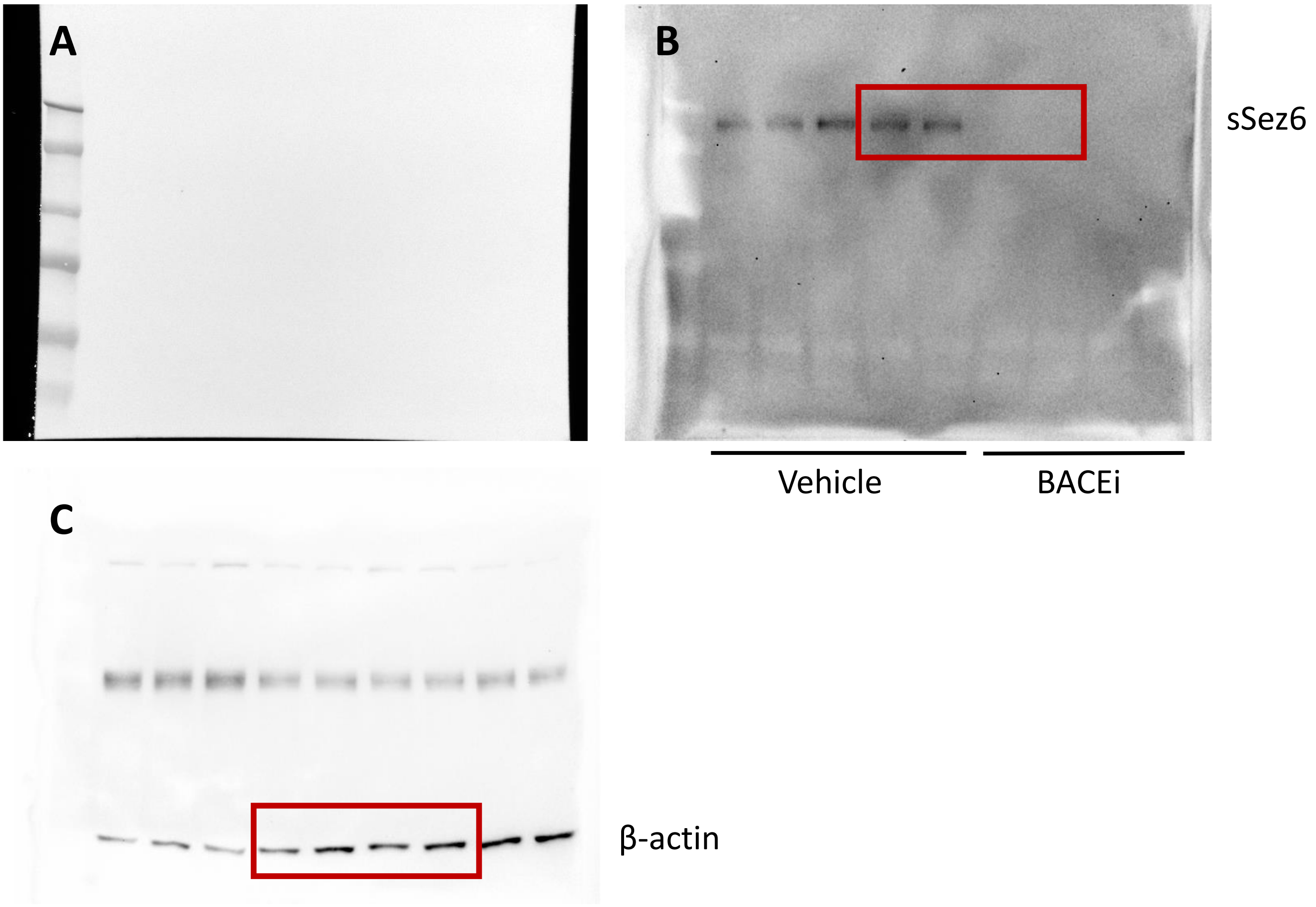

Supplementary Figure 2.

Original Western Blot from Figure 1Ci with molecular weight marker (A), Sez6 ectodomain labelling (B) and β-actin labelling (C).

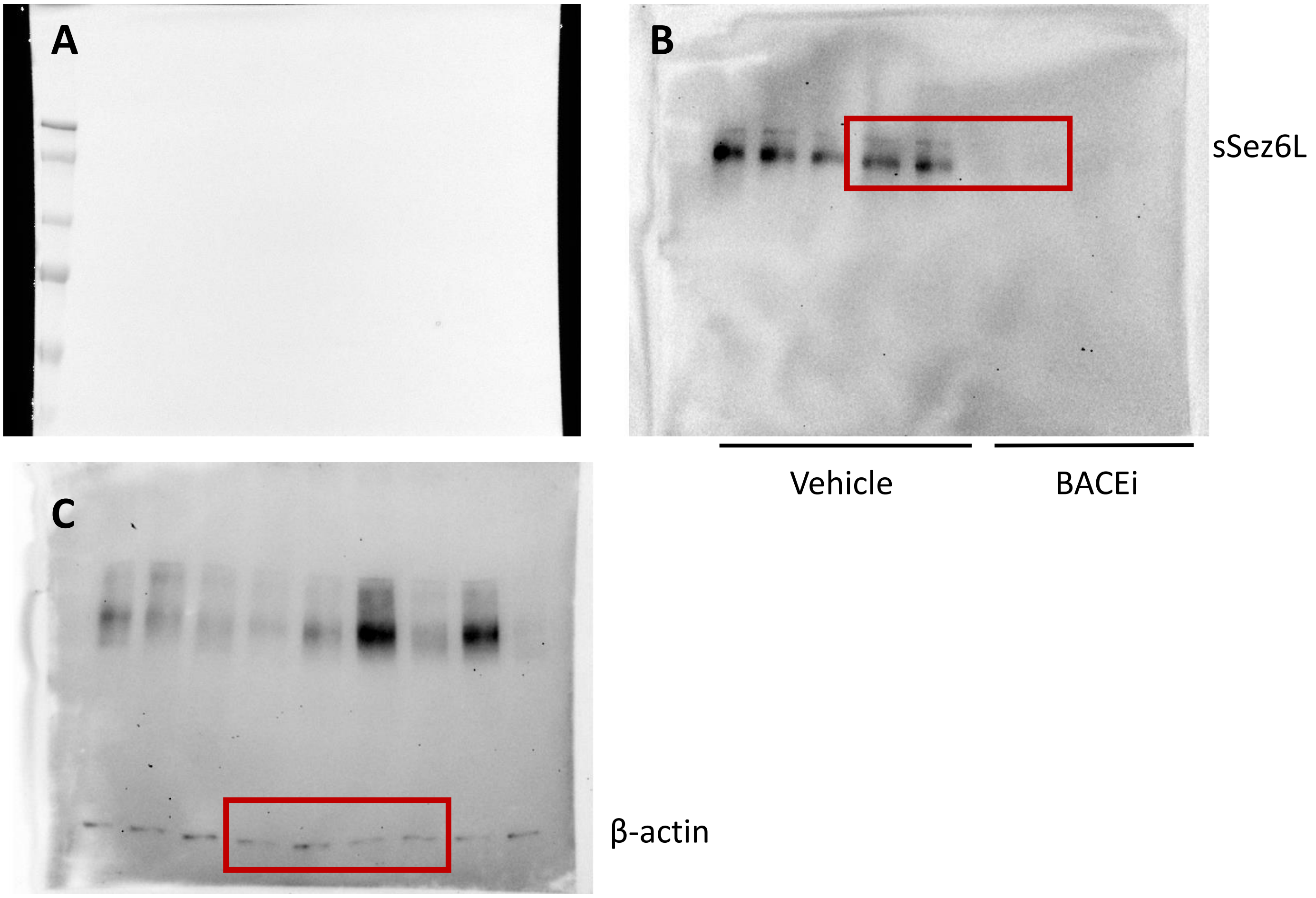

Supplementary Figure 3.

Original Western Blot from Figure 1Ci with molecular weight marker (A), Sez6L ectodomain labelling (B) and β-actin labelling (C).

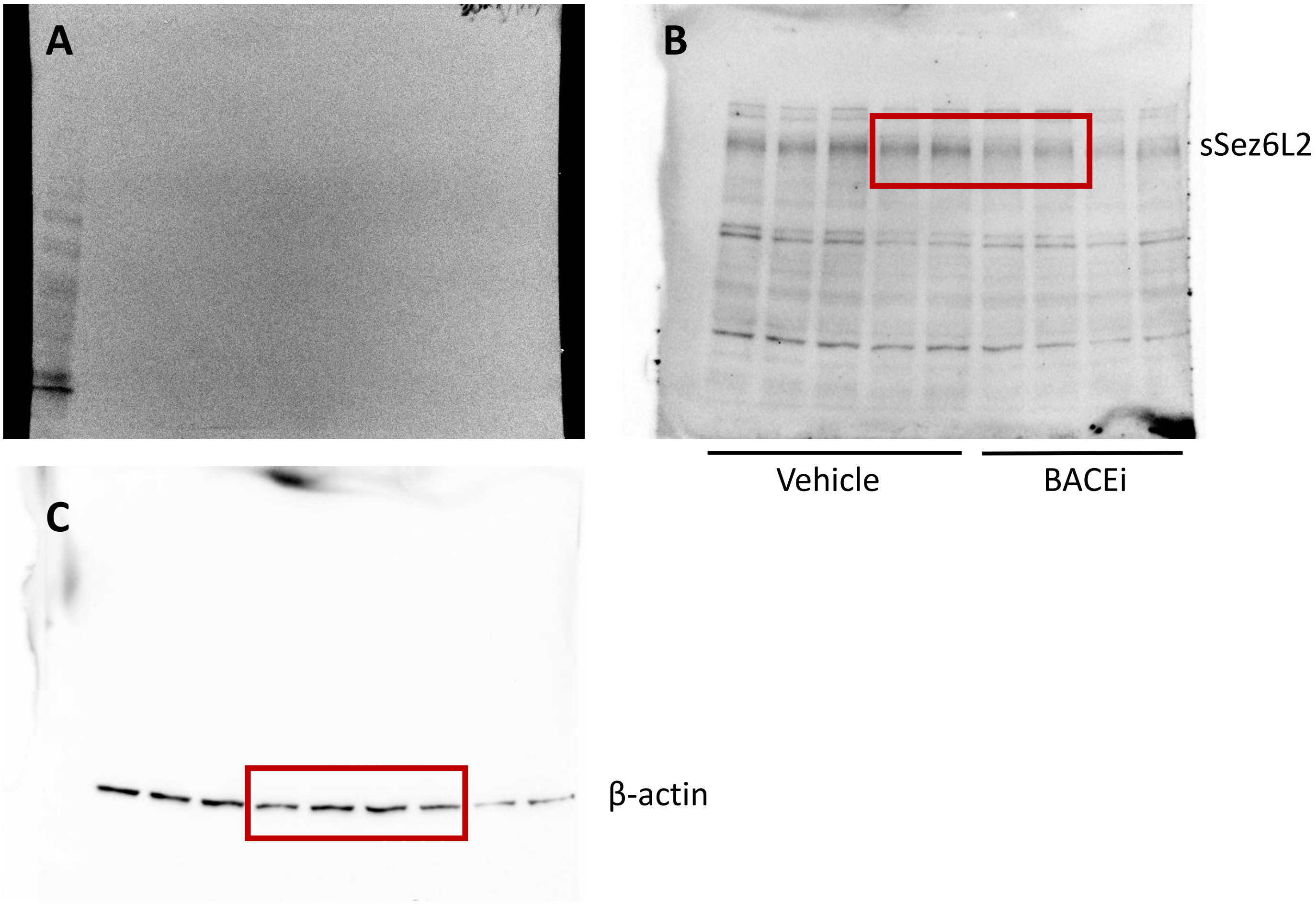

Supplementary Figure 4.

Original Western Blot from Figure 1Ci with molecular weight marker (A), Sez6L2 ectodomain labelling (B) and β-actin labelling (C).

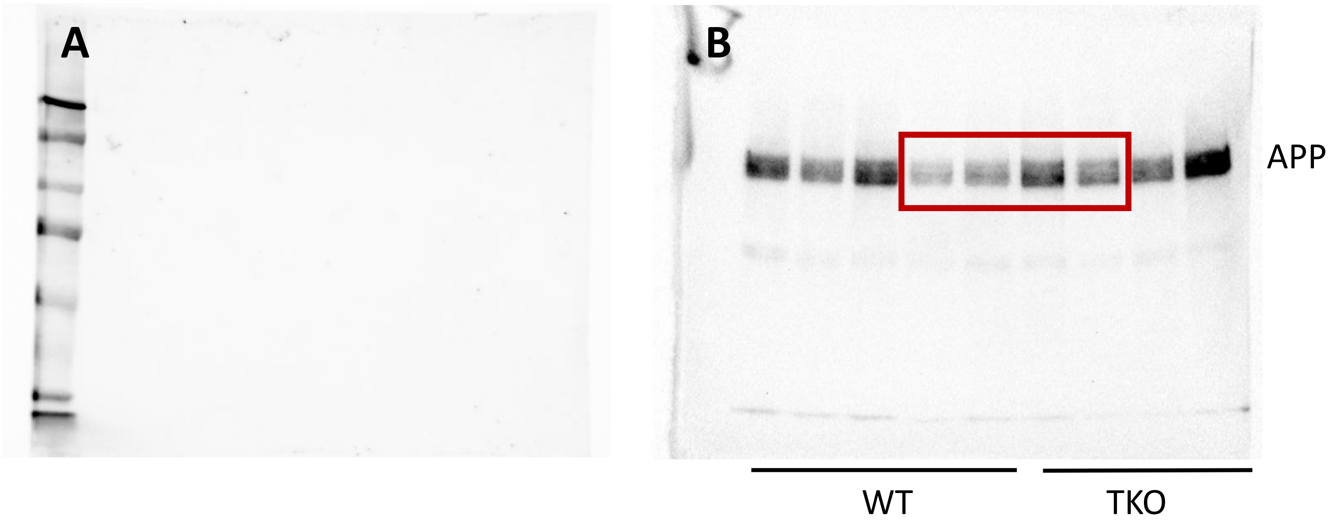

Supplementary Figure 5.

Original Western Blot from Supplementary Figure 1E with molecular weight marker (A) and Amyloid Precursor Protein labelling (B).

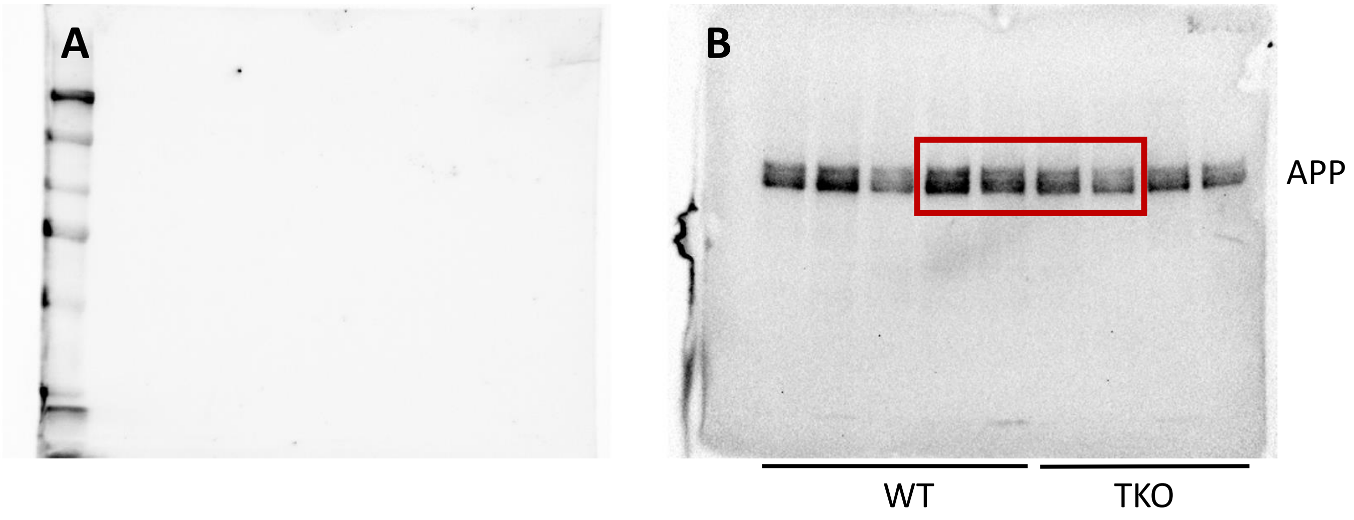

Supplementary Figure 6.

Original Western Blot from Supplementary Figure 1F with molecular weight marker (A) and Amyloid Precursor Protein labelling (B).
